# Maternal Iodine Intake and Neurodevelopment of Offspring: The Japan Environment and Children’s Study

**DOI:** 10.3390/nu14091826

**Published:** 2022-04-27

**Authors:** Aya Hisada, Rieko Takatani, Midori Yamamoto, Hiroko Nakaoka, Kenichi Sakurai, Chisato Mori, the Japan Environment and Children’s Study (JECS) Group

**Affiliations:** 1 Center for Preventive Medical Sciences, Chiba University, 1–33 Yayoi-cho, Inage-ku, Chiba 263-8522, Japan; rieko-takatani@chiba-u.jp (R.T.); midoriy@faculty.chiba-u.jp (M.Y.); hnakaoka@faculty.chiba-u.jp (H.N.); sakuraik@faculty.chiba-u.jp (K.S.); cmori@faculty.chiba-u.jp (C.M.); 2 Department of Bioenvironmental Medicine, Graduate School of Medicine, Chiba University, 1-8-1 Inohana, Chuo-ku, Chiba 260-8670, Japan

**Keywords:** iodine intake, food frequency questionnaire, pregnancy, neurodevelopment, birth cohort study, Ages and Stages Questionnaire Third Edition

## Abstract

Inadequate maternal iodine intake affects thyroid function and may impair fetal brain development. This study investigated the association between maternal iodine intake during pregnancy and neurodevelopmental delay in offspring at 1 and 3 years of age using a nationwide birth cohort: the Japan Environment and Children’s Study. We assessed dietary iodine intake during pregnancy using a food frequency questionnaire and child neurodevelopment using the Japanese translation of the Ages and Stages Questionnaire, Third Edition. The risk of delay (score below the cut-off value) for fine motor domain at 1 year of age was increased in the lowest quintile iodine intake group compared with the fourth quintile iodine intake group. The risk of delay for problem-solving at 1 year of age was increased in the lowest and second quintile iodine intake group and decreased in the highest quintile iodine intake group. The risk of delay for communication, fine motor, problem-solving, and personal–social domains at 3 years of age was increased in the lowest and second quintile iodine intake group compared with the fourth quintile iodine intake group, while the risk of delay for fine motor and problem-solving domains was decreased in the highest quintile iodine intake group. Low iodine intake levels in pregnancy may affect child neurodevelopment.

## 1. Introduction

Iodine is among the constituent elements of thyroid hormones and is essential for thyroid hormone production and neurodevelopment in children. Iodine deficiency is one of the major public health problems, particularly in pregnant women and young children [1]. Iodine deficiency has several adverse effects on growth and development as a result of inadequate production of thyroid hormones [2]. Iodine requirements during pregnancy are elevated because of increased maternal thyroxine production for the transfer of thyroid hormones to the fetus, as fetal thyroid function depends on the mother [2,3]. The Food and Agriculture Organization of United Nations/World Health Organization (WHO) recommend adequate iodine intake during pregnancy to prevent brain damage or thyroid function disorders [4,5]. As efforts are being made globally to reduce iodine deficiency and severe iodine deficiency is becoming uncommon, public health concern has shifted to mild-to-moderate iodine deficiency [6,7]. Some meta-analyses and systematic reviews have indicated the adverse effects of mild-to-moderate iodine deficiency during pregnancy on neurodevelopment in children [8,9,10].

The WHO recommends an iodine intake of 150 µg/day for adolescents and adults and 250 µg/day for pregnant women [11]. The Dietary Reference Intakes for Japanese indicates 170 µg/day as the estimated average requirement (EAR), 240 µg/day as the recommended dietary allowance (RDA), and 2000 µg/day as the tolerable upper intake level for pregnant women [12]. Currently, there is no official recommendation for the use of iodine supplements in pregnant women in Japan. The Japanese population consumes iodine-rich foods, such as seaweed and kelp, on a daily basis [13]. Kelp (60%) and soup stock (30%) were reportedly the major contributors to habitual iodine intake levels [14]. Some studies have reported an estimated average Japanese adult iodine intake of 1000–3000 µg/day [14,15], and habitual iodine intake among Japanese people is sufficient or higher than the tolerable upper intake level, particularly in older generations [14]. However, some reports have indicated that younger Japanese people tend to consume food with low iodine content [16,17]. Therefore, the percentage of people with insufficient iodine intake, such as modern Japanese young people, including those of reproductive age, may not be small. Furthermore, although reports indicate that iodine intake in Japanese pregnant women is sufficient based on urinary concentrations [13,18], 16% of pregnant women are assumed to have low iodine intake [18], raising concerns about the effect of iodine intake levels by modern Japanese pregnant women on child neurodevelopment. In Japan, iodine intake among pregnant women is typically dependent on food rather than supplements. Japanese dietary habits are varied, such that the range of iodine intake is expected to be wide [13]. Thus, we aimed to examine the effects of iodine intake during pregnancy in modern Japanese women on child neurodevelopment by using a nationwide birth cohort study involving nearly 100,000 pregnant women. This study could contribute important data to address the global concern about the impact of iodine intake from food during pregnancy on child neurodevelopment.

## 2. Materials and Methods

### 2.1. Study Design and Participants

This study was conducted based on the Japan Environment and Children’s Study (JECS). Details of the JECS protocol have been described elsewhere [19]. The JECS is an ongoing nationwide prospective birth cohort study that aims to elucidate environmental factors that affect children’s health and development.

The JECS is funded directly by Japan’s Ministry of the Environment and involves collaborations between the Programme Office (National Institute for Environmental Studies), Medical Support Centre (National Center for Child Health and Development), and 15 Regional Centres (Hokkaido, Miyagi, Fukushima, Chiba, Kanagawa, Koshin, Toyama, Aichi, Kyoto, Osaka, Hyogo, Tottori, Kochi, Fukuoka, and South Kyushu/Okinawa). Pregnant women were recruited for this study between January 2011 and March 2014.

The JECS protocol was reviewed and approved by the Ministry of the Environment’s Institutional Review Board on epidemiological studies and the Ethics Committees of all participating institutions. The JECS was conducted following the principles of the Declaration of Helsinki and other nationally valid regulations and guidelines. All participants provided written informed consent.

The present study was based on the jecs-ta-20190930 dataset. From 104,062 fetal records, we excluded cases of miscarriage, preterm birth, stillbirth, multiple births, maternal thyroid disease, maternal thyroid or antithyroid medication use, maternal iodine medication, and iodinated contrast use; we also excluded pairs with missing data on the estimated dietary iodine intake, dietary total energy intake, and Ages and Stages Questionnaire, Third Edition (ASQ-3) data when the child was 1 year of age. The analysis of neurodevelopment at 3 years of age was conducted in children with ASQ-3 data at 1 year of age. In total, 75,249 and 66,604 mother-child pairs with children aged 1 and 3 years, respectively, were enrolled for analysis.

### 2.2. Maternal Iodine Intake and Kelp and Seaweed Intake

The dietary consumption of iodine, as well as kelp and seaweed intake, was determined using the food frequency questionnaire (FFQ) during the second or third trimester. Participants were asked to respond to questions about their dietary intake in the period between awareness of pregnancy and the second or third trimester. The FFQ has been validated in a large-scale epidemiological study in Japanese subjects, and details of the FFQ used in this study have already been reported [20]. The validity of nutrient intake assessment including iodine during pregnancy using a partially modified FFQ has also been reported by Ogawa et al. [21].

The FFQ consists of nine frequency categories and portions for each food and beverage item. The nine frequency categories for each food and beverage item of the FFQ were: “almost never”, “1–3 times per month”, “1–2 times per week”, “3–4 times per week”, “5–6 times per week”, “once per day”, “2–3 times per day”, “4–6 times per day”, and “7 or more times per day.” The standard portion sizes were specified, and the amount of food intake was rated according to the following three categories: “less than half of the standard”, “same as the standard”, and “more than one-and-a-half times the standard.” We also used the energy intake estimated from the FFQ to adjust for iodine intake. A cohort study of pregnant Japanese women found that energy-adjusted iodine intake did not have a significant relationship with dietary records [21]. Abel et al. also compared the residual method with an energy adjustment and the addition of energy intake as a separate covariate, noting that the residual method produces errors at high and low iodine levels [22]. Therefore, we did not use the residual method for energy adjustment in this study but used energy intake as the adjustment variable.

### 2.3. Neurodevelopmental Assessment

For child development, we used data assessed using the Japanese translation of the Ages and Stages Questionnaire, Third Edition (J-ASQ-3). The ASQ-3 used in this study has been validated in Japanese populations [23]. The ASQ-3 consists of 21 age-specific structured questions for children ages 1–66 months and is divided into five domains: communication, gross motor skills, fine motor skills, problem-solving skills, and personal–social skills [24]. The age-appropriate questionnaire was completed by the parent or caregiver. We used data from the ASQ-3 administered to children at 1 year and 3 years of age. The questionnaire comprises 30 questions for each age. The parent responds “yes” (=10), “sometimes” (=5), or “not yet” (=0), resulting in a score of 0–60 for each domain. Developmental delay was defined based on the cut-off values for the J-ASQ-3 scores reported by Mezawa et al. [23].

### 2.4. Statistical Analysis

We categorized participants according to the quintile of the iodine intake or kelp and seaweed intake. We estimated the associations between iodine intake and neurodevelopmental outcomes by multiple binomial logistic regression analysis as a reference for the fourth quintile group of the iodine intake (176–276 µg/day). We chose the fourth group as a reference because it was approximately in the range from the EAR (170 µg/day) to RDA (240 µg/day) for Japanese women. Sensitivity analyses were conducted, additionally adjusting for household income and parity. Sensitivity analyses were also conducted using re-categorical data for iodine intake (<170 µg/day (below the EAR), 170–239 µg/day (from EAR to RDA), 240–2000 µg/day, >2000 µg/day (over the tolerable upper intake level)). We also estimated the association between kelp and seaweed intake and neurodevelopmental outcomes by multiple binomial logistic regression analysis as a reference for the first quintile group of kelp and seaweed intake. Covariates were selected based on fundamental biological attributes or those that are associated with the outcome in previous studies [22,25,26]. All logistic regression analyses were adjusted for the following: maternal age, pre-pregnancy body mass index, maternal education, smoking during pregnancy, drinking during pregnancy, pregnancy-induced hypertension, diabetes or gestational diabetes mellitus, scales for psychological distress (K6: Japanese version of the Kessler 6 scale) [27], child sex, birth weight, the season of birth (two groups: April to September and October to March), folic acid supplementation, eicosapentaenoic acid supplementation, docosahexaenoic acid supplementation, and energy intake. Analyses only included data without missing covariates, as there were few missing values for each variable. The results are reported as odds ratios (ORs) with 95% confidence intervals (CIs). All analyses were conducted using the IBM SPSS Statistics package, version 23 (IBM Corp., Armonk, NY, USA).

## 3. Results

The median estimated iodine intake during pregnancy in this study was 158 µg/day (interquartile range (IQR): 50–226 µg/day). The percentages of pregnant women below the EAR (170 µg/day) and over the tolerable upper intake level (2000 µg/day) of iodine were 56.7% and 0.2%, respectively. The percentages below the cut-off for J-ASQ-3 at 1 year of age were 0.1% for communication, 5.7% for gross motor, 5.8% for fine motor, 5.3% for problem-solving, and 1.2% for personal–social. At 3 years of age, the percentages below the cut-off for J-ASQ-3 were 3.9%, 4.4%, 7.5%, 7.4%, and 3.2%, respectively. Table 1 shows the characteristics of mothers and children according to the quintiles for maternal iodine intake. The lower iodine intake groups had a slightly younger maternal age, lower parity, lower education status of mother and household income, and higher percentage of smoking during pregnancy. There was no difference with regards to gestational hypertension and diabetes according to the estimated iodine intake level.

Table 2 and Table 3 and Supplemental Tables S1 and S2 show the adjusted ORs and crude ORs and 95% CIs for each neurodevelopmental domain at 1 and 3 years of age according to the quintiles for iodine intake during pregnancy, respectively. The risk of delay for fine motor skills at 1 year of age was increased in the lowest compared with the fourth quintile iodine intake group (OR, 1.19; 95% CI 1.07–1.32). The risk of delay for problem-solving skills at 1 year of age was increased in the first and second quintile iodine intake groups (OR, 1.24; 95% CI 1.11–1.38 and OR, 1.18; 95% CI 1.07–1.31, respectively) and decreased in the fifth quintile iodine intake group (OR, 0.89; 95% CI 0.80–0.99) (Table 2). The risk of delay for communication, fine motor, problem-solving, and personal–social domains at 3 years of age was increased in the first and second iodine intake groups. The risk of delay for fine motor and problem-solving domains at 3 years of age was decreased in the fifth quintile iodine intake group (Table 3). We performed sensitivity analyses for the association between ASQ-3 scores and iodine intake by additionally adjusting for household income and parity or using re-categorical data for iodine intake. The associations were similar to the results of the main analyses (Supplemental Tables S3–S6).

Table 4 and Table 5 show the ORs and 95% CIs for each neurodevelopmental domain at 1 and 3 years of age according to the quintiles for kelp and seaweed intake during pregnancy, respectively. The risk of delay for fine motor and problem-solving skills at 1 year of age was decreased in the second, third, and fifth compared with the first quintile in the kelp and seaweed intake group (Table 4). The risk of delay for each domain except for gross motor at 3 years of age was decreased in the second, third, fourth, and fifth quintiles compared with the first quintile in the kelp and seaweed intake group (Table 5).

## 4. Discussion

As expected, the results of this study showed that: (1) low iodine intake from food during pregnancy increased the risk of delay in child neurodevelopment at 1 and 3 years of age, and (2) the number of pregnant women with inadequate iodine intake was not small.

In our study, the fourth quintile of iodine intake (176–276 µg/day) corresponded to the level of the EAR (170 µg/day) and RDA (240 µg/day). Compared to the fourth quintile, the first quintile (<40 µg/day) and the second quintile (41–123 µg/day) groups had an increased risk of delay in some domains of child neurodevelopment at 1 and 3 years of age. This indicates that the number of pregnant women with iodine deficiency in Japan may not be negligible, and the risk of delay in child neurodevelopment may be increased due to insufficient iodine intake. Similar to our study, several studies have investigated iodine intake from food, mainly during the second trimester, and its relationship with neurodevelopment in children. Abel et al. [22,28] investigated the relationship between iodine intake during the first half of pregnancy and neurodevelopment in children at 3 and 8 years of age, excluding iodine supplement takers. The authors reported language delays, behavioral problems, and reduced fine motor skills at 3 years of age and poor language, school performance, and increased likelihood of special education services at 8 years of age in children born to mothers with iodine levels below the intake level of 160 µg/day. In addition, several observational studies on the relationship between iodine levels using urinary concentrations during pregnancy and neurodevelopmental outcomes have been reported [6,10]. Although these results were inconsistent, several studies found a negative association between urinary iodine and cognitive outcomes in children [29,30,31,32].

In our study, the highest quintile iodine intake group (≥277 μg/day, median 465.5 μg/day) was associated with lower risk in some domains. Conversely, Murcia et al. [33] reported an inverse association between maternal iodine intake and motor development in children at 4–5 years of age. Zhou et al. [25] reported that maternal iodine intake in the lowest (<220 µg/day) and highest (≥391 μg/day) quartiles were associated with a lower cognitive-developmental score (Bayley-III score < 1 standard deviation) in 18-month-old children compared with mothers with an iodine intake in the second quartile. In both studies, the iodine intake level in the high iodine group was comparable to that in the present study. However, our results in the highest iodine intake group were not consistent with those reported by Murcia et al. [33] and Zhou et al. [25]

This inconsistency in results could be attributed to the following factors: (1) different sources of iodine intake (from food or supplementation) and (2) goitrogen intake from food. First, in Japan, the major contributors to habitual iodine intake levels are kelp (60%) and soup stock (30%) [14]. In contrast, pregnant women in many other countries obtain iodine from iodine supplements along with food (e.g., milk, yogurt, fish, and iodized salt) [10]. Zhou et al. reported that the rate of iodine supplement takers was 82% [25]. The absorption of inorganic iodide is >90% in healthy adults [34,35]. On the other hand, low iodine bioavailability in kelp (Kombu seaweed) has been estimated in vitro [36]. Combet et al. also reported lower bioavailability of seaweed iodine (33%, IQR: 23–46%) than bioavailability from potassium iodide supplements (59%, IQR: 46–74%) [37]. Namely, high estimated iodine intake from kelp or seaweed may not lead to adverse effects on child development due to low iodine absorption from kelp. This is supported by the finding in this study that the risk of developmental delay in children was reduced in groups with high intake of kelp and seaweed. Second, Japanese people may usually consume iodine with goitrogens (i.e., soy isoflavones). Soy isoflavones inhibit incorporation of iodine into the thyroid hormone [38,39,40]. Japanese food commonly includes soy-based products such as tofu, miso, natto, and soy sources, and these foods are often eaten with food containing iodine (e.g., seaweed, kelp soup). In fact, a dietary record survey indicated that the typical dietary habit in Japan includes a high consumption of soy isoflavones [41]. Thus, the intake of soy may possibly suppress the effects of excessive iodine intake on child neurodevelopment.

Furthermore, the increased risk of neurodevelopmental delay in low iodine intake groups was more pronounced at 3 years of age than at 1 year of age. The reason for this may be the lower validity of the ASQ-3 at 1 year of age than at 3 years of age [23]. Previous studies in Europe and Australia reported an association between iodine intake during pregnancy and child neurodevelopmental outcomes in offspring between 6 months and 10 years of age [10]. Thus, inadequate iodine nutrition during pregnancy in Japanese women may affect children’s neurodevelopment, and these effects may persist into childhood. Further studies are needed to examine this association.

In our study, the iodine intake level was low compared with previous studies of pregnant Japanese women; 56.7% of pregnant women had inadequate iodine intake (below EAR, 170 μg/day). One reason for this difference could be an underestimation of iodine intake from the FFQ. The FFQ used refers to the Standard Tables of Food Composition in Japan 2010 [42]. However, it is possible that iodine was not sufficiently accounted for in the FFQ because iodine was an additional nutrient item for the revised food composition table [20] and the content containing iodine included only 515 of the 1878 food items [14]; therefore, estimated iodine intake may not have been sufficiently evaluated. The second reason could be the possibility of reduced iodine intake among younger generations of pregnant women due to an increase in the Western diet [43]. Katagiri et al. reported that people who do not eat a traditional Japanese diet consume significantly low amounts of iodine, and this pattern was mainly observed in younger participants [16]. In this study, iodine intake tended to be lower with younger age. The WHO recommends an iodine intake of 250 μg/day for pregnant and lactating women, and pregnant women with iodine deficiency are recommended iodine-containing foods such as iodized salt, iodine supplements, or iodine-fortified foods [11,44]. In Japan, iodine is generally not added to commercially available salt, and there is no official recommendation for pregnant women to take iodine supplements in Japan because consumption of iodine-rich seaweed and the use of soup stock made from kelp is considered to provide sufficient levels of iodine. There were concerns about hypothyroidism [45,46] and other conditions in newborns due to excessive iodine intake of pregnant women in Japan. However, the number of pregnant women with inadequate iodine intake was not small, and low iodine intake could increase the risk of neurodevelopmental delay of children in this study. Our study results may suggest the need for pregnant women to supplement iodine.

One of the strengths of this study is that it used data from a nationwide cohort study, enrolling nearly 100,000 participants in Japan [19]. The use of a large cohort enabled the analysis of a wide range of iodine intake levels from food. This study contributes important data to address the global concern about the impact of iodine intake from food during pregnancy on child neurodevelopment. In addition, using the ASQ-3, a validated neurodevelopmental assessment tool used worldwide [24], made it possible to conduct a large-scale neurodevelopmental assessment. Lastly, the use of prospective cohort study data allowed us to assess neurodevelopment over time in children up to the age of 3.

Some limitations of this study should be noted. First, as mentioned earlier, iodine intake assessed using FFQ may lead to an underestimation of the iodine intake level. However, a validation study in pregnant women, using a partially modified version of the FFQ used in the JECS, reported a significant association between the FFQ and 3-day dietary records [21]. Therefore, iodine intake estimated from the FFQ may be a reliable indicator of iodine intake trends. Second, there may be confounding factors that could not be measured (e.g., maternal IQ and genomic factors). Third, there is insufficient consideration of the effects of mild hypothyroidism on child neurodevelopment. Recent reports have indicated that minor imbalances of thyroid hormone may impact offspring neurodevelopment [47,48]. We could not exclude mild thyroid function deficiency because thyroid hormones during pregnancy were not measured in this study. Therefore, we only excluded maternal thyroid disease, and thyroid and antithyroid medication use due to exclude effects on neurodevelopment from maternal abnormal thyroid function. Fourth, we adjusted all multiple binomial logistic regression analyses for folic acid, eicosapentaenoic acid, and docosahexaenoic acid supplementation, as previous studies found that these specifically affect neurodevelopment [49,50,51]. However, regarding nutrients other than iodine, a recent review has shown that inadequate nutrient (vitamins, micronutrients, macronutrients) intake during pregnancy was associated with brain defects, an increased risk of abnormal behavior, neurodevelopmental disorders, altered cognition, and visual impairment and motor deficits [52]. It may be necessary to consider multiple markers of nutritional status in the future. Fifth, the FFQ and ASQ-3 are self-administered questionnaires, which can lead to risks such as recall bias, social desirability bias, and misinterpretation. Lastly, this study surveyed Japanese women who generally consume seaweed more than other populations in different countries. Therefore, generalization of these findings should be approached with caution.

## 5. Conclusions

In conclusion, this study found that low iodine intake among pregnant women might affect child neurodevelopment at 1 and 3 years of age. Recently, as the proportion of pregnant women in Japan with inadequate iodine intake is considered not to be negligible, intake of iodine may need to be recommended for pregnant women in Japan. It is important to collate data on the relationship between urinary iodine concentration, dietary intake, and child development and investigate the optimal iodine dietary intake for neurodevelopment. Follow-up of the JECS is scheduled up until the children reach 13 years of age. We consider that it is crucial to continue investigating the effects of iodine intake during pregnancy on neurodevelopment.

## Figures and Tables

**Table 1:** Participant characteristics according to the quintiles for iodine intake during pregnancy.

	Quintile for Iodine Intake				
	1 (≤40 µg/Day)	2 (41–123 µg/Day)	3 (124–175 µg/Day)	4 (176–276 µg/Day)	5 (≥277 µg/Day)
*n* = 75,249	15,349	14,772	15,029	15,064	15,035
Iodine intake, µg/day (median)	22.5	71.0	155	209	465.5
Maternal age, years (mean (SD))	29.9 (5.2)	31.1 (5.0)	31.4 (4.8)	31.9 (4.6)	32.0 (4.7)
BMI (mean (SD))	21.4 (3.5)	21.1 (3.2)	21.1 (3.2)	21.0 (3.0)	21.1 (3.1)
Parity (%)					
0	7167 (48)	6324 (44)	5977 (40.8)	5502 (37.3)	5661 (38.5)
1	5242 (35.1)	5395 (37.5)	5780 (39.4)	6190 (42.0)	5887 (40.1)
≥2	2536 (17)	2668 (18.5)	2895 (19.8)	3060 (20.7)	3138 (21.4)
Marital status unmarried/married (%)	988/14,223 (6.5/93.5)	621/14,033 (4.2/95.8)	507/14,440 (3.4/96.6)	395/14,561 (2.6/97.4)	498/14,432 (3.3/96.7)
Education status of mother (%)					
<10 years	1029 (6.8)	638 (4.3)	553 (3.7)	407 (2.7)	441 (2.9)
10–12 years	5818 (38.3)	4647 (31.6)	4492 (30.0)	3951 (26.3)	3957 (26.4)
13–16 years	8176 (53.8)	9206 (62.5)	9709 (64.9)	10,422 (69.3)	10,288 (68.6)
≥17 years	167 (1.1)	236 (1.6)	212 (1.4)	254 (1.7)	305 (2.0)
Household income					
<2,000,000 yen	1154 (8.2)	762 (5.5)	601 (4.3)	489 (3.4)	592 (4.2)
2,000,000–4,000,000	5694 (40.7)	4855 (35.2)	4832 (34.3)	4437 (31.1)	4263 (30.2)
4,000,000–6,000,000	4227 (30.2)	4660 (33.8)	4749 (33.7)	5015 (35.1)	4885 (34.6)
6,000,000–8,000,000	1847 (13.2)	2093 (15.2)	2328 (16.5)	2521 (17.7)	2567 (18.2)
8,000,000–10,000,000	677 (4.8)	871 (6.3)	987 (7.0)	1090 (7.6)	1052 (7.5)
≥10,000,000	399 (2.9)	543 (3.9)	597 (4.2)	720 (5.0)	758 (5.4)
Maternal smoking (%)					
never smoked	8211 (54.0)	8644 (58.9)	9067 (60.7)	9391 (62.7)	9143 (61.2)
quit before pregnancy	3450 (22.7)	3447 (23.5)	3579 (24.0)	3644 (24.3)	3750 (25.1)
quit during pregnancy	2696 (17.7)	1950 (13.3)	1799 (12.1)	1517 (10.1)	1616 (10.8)
smoking during pregnancy	845 (5.6)	626 (4.3)	483 (3.2)	421 (2.8)	437 (2.9)
Maternal drinking (%)					
never drank	5198 (34.3)	5043 (34.4)	5045 (33.8)	4999 (33.4)	4871 (32.6)
quit before pregnancy	2456 (16.2)	2425 (16.5)	2504 (16.8)	2548 (17.0)	2727 (18.2)
quit during pregnancy	7099 (46.8)	6782 (46.2)	7040 (47.1)	6987 (46.6)	6925 (46.3)
drinking during pregnancy	411 (2.7)	418 (2.8)	354 (2.4)	455 (3.0)	437 (2.9)
Hypertension no/yes (%)	14,926/423 (97.2/2.8)	14,389/383 (97.4/2.6)	14,642/387 (97.4/2.6)	14,719/345 (97.7/2.3)	14,624/411 (97.3/2.7)
Diabetes or gestational diabetes mellitus no/yes (%)	14,917/432 (97.2/2.8)	14,360/412 (97.2/2.8)	14,574/455 (97.0/3.0)	14,627/437 (97.1/2.9)	14,500/535 (96.4/3.6)
K6 (mean (SD))	2.8 (3.8)	2.9 (3.7)	2.6 (3.4)	2.7 (3.4)	2.8 (3.6)
Child’s sex: boys/girls (%)	7821/7528 (51.0/49.0)	7511/7261 (50.8/49.2)	7663/7366 (51.0/49.0)	7698/7366 (51.1/48.9)	7630/7405 (50.7/49.3)
Birth weight, g (mean (SD))	3053 (367)	3061 (366)	3062 (367)	3071 (367)	3064 (365)
Birth length, cm (mean (SD))	49.1 (1.9)	49.1 (2.0)	49.1 (1.9)	49.2 (1.9)	49.1 (1.9)
Birth season spring-summer/autumn-winter (%)	7937/7412 (51.7/48.3)	7817/6955 (52.9/47.1)	7775/7254 (51.7/48.3)	8002/7062 (53.1/46.9)	8116/6919 (54.0/46.0)

**Table 2:** Adjusted ORs and 95% CIs for child neurodevelopmental delay at 1 year of age based on maternal iodine intake in multiple binomial logistic regression analysis.

	Quintile for Iodine Intake								
	1		2		3		4	5	
	OR	95% CI	OR	95% CI	OR	95% CI	OR	OR	95% CI
Communication	0.68	0.35, 1.33	0.76	0.41, 1.40	0.76	0.41, 1.41	1	0.79	0.44, 1.44
Gross Motor	0.94	0.84, 1.04	0.98	0.89, 1.09	0.98	0.89, 1.08	1	0.98	0.89, 1.08
Fine Motor	**1.19**	**1.07, 1.32**	1.07	0.97, 1.18	1.07	0.97, 1.18	1	0.92	0.83, 1.02
Problem-Solving	**1.24**	**1.11, 1.38**	**1.18**	**1.07, 1.31**	1.07	0.96, 1.19	1	**0.89**	**0.80, 0.99**
Personal–Social	0.82	0.66, 1.02	**0.80**	**0.65, 0.99**	0.88	0.71, 1.07	1	0.87	0.71, 1.07

The models were adjusted for maternal age, pre-pregnancy body mass index, maternal education, smoking during pregnancy, drinking during pregnancy, gestational hypertension, diabetes or gestational diabetes mellitus, scales for psychological distress (K6: Japanese version of the Kessler 6 scale), child sex, birth weight, the season of birth (two groups: April to September and October to March), folic acid supplementation, eicosapentaenoic acid supplementation, docosahexaenoic acid supplementation, and energy intake. OR: odds ratio; CI: confidence interval; bold font: increasing or decreasing tendency of neurodevelopmental delay risk.

**Table 3:** Adjusted ORs and 95% CIs for child neurodevelopmental delay at 3 years of age based on maternal iodine intake in multiple binomial logistic regression analysis.

	Quintile for Iodine Intake								
	1		2		3		4	5	
	OR	95% CI	OR	95% CI	OR	95% CI	OR	OR	95% CI
Communication	**1.18**	**1.03, 1.34**	**1.17**	**1.03, 1.32**	0.99	0.87, 1.13	1	0.96	0.84, 1.09
Gross Motor	1.07	0.95, 1.22	**1.13**	**1.01, 1.27**	0.97	0.86, 1.10	1	0.92	0.81, 1.04
Fine Motor	**1.19**	**1.08, 1.31**	**1.14**	**1.04, 1.26**	1.01	0.91, 1.11	1	**0.88**	**0.80, 0.97**
Problem-Solving	**1.10**	**1.00, 1.22**	**1.12**	**1.02, 1.23**	0.92	0.83, 1.01	1	**0.88**	**0.79, 0.96**
Personal–Social	**1.14**	**0.99, 1.32**	**1.16**	**1.01, 1.34**	0.93	0.80, 1.08	1	0.98	0.85, 1.13

The models were adjusted for maternal age, pre-pregnancy body mass index, maternal education, smoking during pregnancy, drinking during pregnancy, gestational hypertension, diabetes or gestational diabetes mellitus, scales for psychological distress (K6: Japanese version of the Kessler 6 scale), child sex, birth weight, the season of birth (two groups: April to September and October to March), folic acid supplementation, eicosapentaenoic acid supplementation, docosahexaenoic acid supplementation, and energy intake. OR: odds ratio; CI: confidence interval; bold font: increasing or decreasing tendency of neurodevelopmental delay risk.

**Table 4:** Adjusted ORs and 95% CIs for child neurodevelopmental delay at 1 year of age based on maternal kelp and seaweed intake in multiple binomial logistic regression analysis.

	Quintile for Kelp and Seaweed Intake								
	1	2		3		4		5	
	OR	OR	95% CI	OR	95% CI	OR	95% CI	OR	95% CI
Communication	1	1.19	0.65, 2.16	1.07	0.61, 1.89	1.01	0.23, 4.34	0.89	0.45, 1.76
Gross Motor	1	1.00	0.91, 1.10	0.96	0.88, 1.04	1.11	0.89, 1.38	0.93	0.84, 1.03
Fine Motor	1	**0.87**	**0.79, 0.95**	**0.86**	**0.79, 0.93**	0.93	0.74, 1.17	**0.84**	**0.76, 0.93**
Problem-Solving	1	**0.81**	**0.74, 0.89**	**0.83**	**0.76, 0.91**	0.91	0.72, 1.15	**0.79**	**0.71, 0.88**
Personal–Social	1	1.01	0.83, 1.23	1.10	0.92, 1.32	0.83	0.48, 1.41	0.92	0.74, 1.15

The models were adjusted for maternal age, pre-pregnancy body mass index, maternal education, smoking during pregnancy, drinking during pregnancy, gestational hypertension, diabetes or gestational diabetes mellitus, scales for psychological distress (K6: Japanese version of the Kessler 6 scale), child sex, birth weight, the season of birth (two groups: April to September and October to March), folic acid supplementation, eicosapentaenoic acid supplementation, docosahexaenoic acid supplementation, and energy intake. OR: odds ratio; CI: confidence interval; bold font: increasing or decreasing tendency of neurodevelopmental delay risk.

**Table 5:** Adjusted ORs and 95% CIs for child neurodevelopmental delay at 3 years of age based on maternal kelp and seaweed intake in multiple binomial logistic regression analysis.

	Quintile for Kelp and Seaweed Intake								
	1	2		3		4		5	
	OR	OR	95%CI	OR	95%CI	OR	95%CI	OR	95%CI
Communication	1	**0.88**	**0.79, 0.99**	**0.87**	**0.78, 0.97**	0.73	0.53, 1.01	**0.86**	**0.76, 0.98**
Gross Motor	1	0.90	0.80, 1.00	0.91	0.82, 1.01	0.98	0.75, 1.28	**0.88**	**0.78, 0.99**
Fine Motor	1	**0.86**	**0.79, 0.94**	**0.78**	**0.71, 0.84**	**0.79**	**0.63, 0.99**	**0.74**	**0.67, 0.82**
Problem-Solving	1	0.93	0.85, 1.01	**0.86**	**0.79, 0.93**	**0.77**	**0.61, 0.97**	**0.84**	**0.76, 0.92**
Personal–Social	1	0.91	0.80, 1.04	**0.88**	**0.78, 1.00**	0.97	0.71, 1.33	**0.81**	**0.70, 0.94**

The models were adjusted for maternal age, pre-pregnancy body mass index, maternal education, smoking during pregnancy, drinking during pregnancy, gestational hypertension, diabetes or gestational diabetes mellitus, scales for psychological distress (K6: Japanese version of the Kessler 6 scale), child sex, birth weight, season of birth (two groups: April to September and October to March), folic acid supplementation, eicosapentaenoic acid supplementation, docosahexaenoic acid supplementation, and energy intake. OR: odds ratio; CI: confidence interval; bold font: increasing or decreasing tendency of neurodevelopmental delay risk.

## Data Availability

Data are unsuitable for public deposition due to the ethical restrictions and the legal framework of Japan. It is prohibited by the Act on the Protection of Personal Information (Act No. 57 of 30 May 2003, amendment on 9 September 2015) to publicly deposit data containing personal information. Ethical Guidelines for Medical and Health Research Involving Human Subjects enforced by the Japan Ministry of Education, Culture, Sports, Science and Technology and the Ministry of Health, Labour, and Welfare also restrict the open sharing of epidemiological data. All inquiries about access to data should be sent to: jecs-en@nies.go.jp. The person responsible for handling enquiries sent to this e-mail address is Dr. Shoji F. Nakayama, JECS Programme Office, National Institute for Environmental Studies.

## Supplementary Materials

The following supporting information can be downloaded at: https://www.mdpi.com/article/10.3390/nu14091826/s1, Table S1: Crude ORs and 95% CIs for child neurodevelopmental delay at 1 year of age based on maternal iodine intake; Table S2: Crude ORs and 95% CIs for child neurodevelopmental delay at 3 years of age based on maternal iodine intake; Table S3: Adjusted ORs and 95% CIs for child neurodevelopmental delay at 1 year of age based on maternal iodine intake; Table S4: Adjusted ORs and 95% CIs for child neurodevelopmental delay at 3 years of age based on maternal iodine intake; Table S5: Adjusted ORs and 95% CIs for child neurodevelopmental delay at 1 year of age based on maternal iodine intake; Table S6: Adjusted ORs and 95% CIs for child neurodevelopmental delay at 3 years of age based on maternal iodine intake.
