# Supplementary material for: Maternal Iodine Intake and Neurodevelopment of Offspring: The Japan Environment and Children’s Study
Source: Nutrients. 2022 Apr 27;14(9):1826. doi: 10.3390/nu14091826 (PMC9105063; doi:10.3390/nu14091826)
Supplement: Supplementary file 1 [file nutrients-14-01826-s001.zip › nutrients-1660997-supplementary.pdf]

nutrients

MDPI

Supplementary Materials

Maternal Iodine Intake and Neurodevelopment of Offspring: The Japan Environment and Children's Study

Aya Hisada [1,*], Rieko Takatani [1], Midori Yamamoto [1], Hiroko Nakaoka [1], Kenichi Sakurai [1], Chisato Mori [1,2] and the Japan Environment and Children's Study (JECS) Group [†]

Table S1. Crude ORs and 95% CIs for child neurodevelopmental delay at 1 year of age based on maternal iodine intake.

	Quintile for iodine intake								
	1		2		3		4	5	
	OR	95%CI	OR	95%CI	OR	95%CI	OR	OR	95%CI
Communication	0.63	0.34, 1.18	0.73	0.40, 1.35	0.72	0.39, 1.32	1	0.88	0.50, 1.56
Gross Motor	0.86	0.78, 0.95	0.95	0.86, 1.05	0.95	0.87, 1.05	1	0.98	0.89, 1.07
Fine Motor	1.11	1.01, 1.22	1.06	0.96, 1.17	1.05	0.95, 1.15	1	0.93	0.84, 1.02
Problem-Solving	1.15	1.04, 1.27	1.17	1.06, 1.29	1.04	0.94, 1.15	1	0.90	0.81, 1.00
Personal-Social	0.78	0.64, 0.96	0.82	0.67, 1.00	0.89	0.73, 1.08	1	0.89	0.73, 1.08

OR, odds ratio; CI, confidence interval.

Table S2. Crude ORs and 95% CIs for child neurodevelopmental delay at 3 years of age based on maternal iodine intake.

	**Quintile for iodine intake**								
	**1**		**2**		**3**		**4**	**5**	
	**OR**	**95%CI**	**OR**	**95%CI**	**OR**	**95%CI**	**OR**	**OR**	**95%CI**
Communication	1.16	1.02, 1.31	1.17	1.03, 1.32	0.97	0.85, 1.10	1	0.98	0.86, 1.11
Gross Motor	1.01	0.9, 1.14	1.12	1.00, 1.25	0.95	0.84, 1.07	1	0.94	0.84, 1.06
Fine Motor	1.15	1.05, 1.26	1.15	1.05, 1.26	0.97	0.88, 1.07	1	0.92	0.83, 1.01
Problem-Solving	1.08	0.98, 1.18	1.13	1.03, 1.24	0.90	0.82, 0.99	1	0.91	0.83, 1.00
Personal-Social	1.12	0.98, 1.28	1.18	1.03, 1.34	0.90	0.78, 1.04	1	1.00	0.87, 1.15

OR, odds ratio; CI, confidence interval.

Table S3. Adjusted ORs and 95% CIs for child neurodevelopmental delay at 1 year of age based on maternal iodine intake.

	Quintile for iodine intake								
	1		2		3		4	5	
	**OR**	**95%CI**	**OR**	**95%CI**	**OR**	**95%CI**	**OR**	**OR**	**95%CI**
Communication	0.66	0.33, 1.32	0.75	0.40, 1.41	0.74	0.39, 1.39	1	0.84	0.46, 1.53
Gross Motor	0.93	0.84, 1.04	0.96	0.86, 1.06	0.97	0.88, 1.08	1	0.96	0.87, 1.07
Fine Motor	1.15	1.04, 1.29	1.06	0.96, 1.17	1.02	0.92, 1.13	1	0.91	0.82, 1.01
Problem-Solving	1.21	1.08, 1.35	1.15	1.03, 1.28	1.05	0.94, 1.18	1	0.87	0.78, 0.98
Personal-Social	0.84	0.67, 1.06	0.79	0.64, 0.99	0.84	0.68, 1.04	1	0.90	0.73, 1.11

The models were adjusted for maternal age, pre-pregnancy body mass index, maternal education, smoking during pregnancy, drinking during pregnancy, gestational hypertension, diabetes or gestational diabetes mellitus, scales for psychological distress (K6: Japanese version of the Kessler 6 scale), child sex, birth weight, the season of birth (two groups: April to September and October to March), folic acid supplementation, eicosapentaenoic acid supplementation, docosahexaenoic acid supplementation, energy intake, household income and parity.

OR, odds ratio; CI, confidence interval.

Table S4. Adjusted ORs and 95% CIs for child neurodevelopmental delay at 3 years of age based on maternal iodine intake.

	Quintile for iodine intake								
	1		2		3		4	5	
	OR	95%CI	OR	95%CI	OR	95%CI	OR	OR	95%CI
Communication	1.15	1.00, 1.32	1.16	1.01, 1.32	0.98	0.85, 1.12	1	0.92	0.80, 1.06
Gross Motor	1.05	0.92, 1.19	1.08	0.96, 1.22	0.95	0.84, 1.08	1	0.88	0.77, 1.00
Fine Motor	1.15	1.04, 1.27	1.11	1.01, 1.23	0.99	0.89, 1.09	1	0.87	0.79, 0.97
Problem-Solving	1.08	0.98, 1.20	1.10	1.00, 1.22	0.91	0.83, 1.01	1	0.87	0.78, 0.96
Personal-Social	1.11	0.95, 1.29	1.17	1.02, 1.36	0.91	0.78, 1.06	1	0.98	0.84, 1.13

The models were adjusted for maternal age, pre-pregnancy body mass index, maternal education, smoking during pregnancy, drinking during pregnancy, gestational hypertension, diabetes or gestational diabetes mellitus, scales for psychological distress (K6: Japanese version of the Kessler 6 scale), child sex, birth weight, the season of birth (two groups: April to September and October to March), folic acid supplementation, eicosapentaenoic acid supplementation, docosahexaenoic acid supplementation, energy intake, household income and parity.

OR, odds ratio; CI, confidence interval.

Table S5. Adjusted ORs and 95% CIs for child neurodevelopmental delay at 1 year of age based on maternal iodine intake.

	< 170 μg/day		170–239 μg/day	240–2000 μg/day		> 2000 μg/day	
	OR	95%CI	OR	OR	95%CI	OR	95%CI
Communication	0.84	0.50, 1.41	1	1.02	0.57, 1.82	-	-
Gross Motor	0.98	0.90, 1.06	1	0.98	0.89, 1.07	1.32	0.63, 2.76
Fine Motor	1.10	1.01, 1.19	1	0.94	0.85, 1.03	1.34	0.64, 2.80
Problem-Solving	1.18	1.08, 1.29	1	0.93	0.83, 1.03	0.70	0.25, 1.93
Personal-Social	0.83	0.70, 0.98	1	0.90	0.74, 1.09	1.33	0.32, 5.54

The models were adjusted for maternal age, pre-pregnancy body mass index, maternal education, smoking during pregnancy, drinking during pregnancy, gestational hypertension, diabetes or gestational diabetes mellitus, scales for psychological distress (K6: Japanese version of the Kessler 6 scale), child sex, birth weight, the season of birth (two groups: April to September and October to March), folic acid supplementation, eicosapentaenoic acid supplementation, docosahexaenoic acid supplementation, and energy intake.

OR, odds ratio; CI, confidence interval.

Table S6. Adjusted ORs and 95% CIs for child neurodevelopmental delay at 3 years of age based on maternal iodine intake.

	< 170 μg/day		170–239 μg/day	240–2000 μg/day		> 2000 μg/day	
	OR	95%CI	OR	OR	95%CI	OR	95%CI
Communication	1.12	1.01, 1.25	1	0.96	0.84, 1.08	1.60	0.70, 3.65
Gross Motor	1.07	0.97, 1.18	1	0.92	0.82, 1.04	1.76	0.83, 3.73
Fine Motor	1.11	1.02, 1.20	1	0.89	0.81, 0.97	1.17	0.59, 2.32
Problem-Solving	1.10	1.02, 1.20	1	0.95	0.86, 1.04	0.90	0.42, 1.93
Personal-Social	1.11	0.98, 1.24	1	0.98	0.85, 1.12	1.40	0.54, 3.63

The models were adjusted for maternal age, pre-pregnancy body mass index, maternal education, smoking during pregnancy, drinking during pregnancy, gestational hypertension, diabetes or gestational diabetes mellitus, scales for psychological distress (K6: Japanese version of the Kessler 6 scale), child sex, birth weight, the season of birth (two groups: April to September and October to March), folic acid supplementation, eicosapentaenoic acid supplementation, docosahexaenoic acid supplementation, and energy intake.

OR, odds ratio; CI, confidence interval.
